# Unsuccessful Practitioners

**Published:** 1875-10

**Authors:** 


### MISCELLANEOUS.

### "Unsuccessful Practitioners."

TO THE EDITOR OF THE MEDICAL RECORD.

MR. EDITOR:—In the struggle for existence somebody is always getting pushed to the wall. It always has been so, and doubtless will always continue to be so. Some of these unfortunates drop out of the crush quietly; others signify their discomfiture by loud wails and lamentations, like your correspondent "Diploma." It matters little in what particular line this struggle has been, whether literature, science, art, trade, commerce, or manufacture, the burden of the wail of unsuccessful men is nearly always the same, viz., *that* particular calling is too crowded, and the great public too unappreciative; and those who have attained success have stumbled upon it, fallen into it, or been conducted to it by the wealth and influence of others. Unsuccessful men have ever modestly disclaimed any responsibility for their own failures.

Such being the case—and I believe you will concede the truth of my statements—I should not have taken upon myself to criticise the letter of your correspondent, did you not in your editorial upon the subject allude to it as "a very interesting, instructive, and suggestive letter," vouch for "the facts which he presents," favor holding the medical colleges to an accountability for overcrowding the profession, and inquire if "in the confusion of struggling multitudes, is there an even chance that the truly meritorious one will always succeed?"

Now, Mr. Editor, I am in no way personally connected with any of the medical colleges, nor interested in them save as institutions which have the training of our young men, and thus give character to our profession. On this account alone I cannot calmly bear to have them maligned.

Furthermore, I have always believed that honest and intelligent perseverance to *any legitimate* business will certainly bring success, unavoidable accidents alone excepted, of course. I believe it none the less firmly since reading "Diploma's" letter.

In the first place, *Is* the profession overcrowded? "Diploma" proves it to his own satisfaction both by statistics and his own experience. But then, anything can be proved by statistics or by personal experience. Personal experience has time and again proved conclusively the existence of ghosts and hobgoblins, has time and again proved insurmountable obstacles in the path of success; yet the great mass of mankind still doubt the existence of the former, and are quite unmindful of the latter. Success still comes to those who work for it, none the less than it did fifty or one hundred years ago.

That now and then a man fails to succeed in the practice of medicine proves nothing as to the vocation being overcrowded. For one, I do not believe in this alleged overcrowding, but, on the contrary, maintain that the profession offers excellent promise of a livelihood and more, to young men of perseverance and ability.

I do not say that they will obtain it in the way your correspondent has pursued. Upon his own showing, I think the responsibility for his failure can rest upon no one but himself. He had the best of advantages, and by all natural laws should have won in the battle for existence. But, with all the advantage which culture could give, on one occasion he was worsted by "a pair of pestilent, vulgar, and ill-educated quacks." Tell me of what advantage would it be to him to have the standard of medical education raised, when even the ill-educated quack has more *moral* control over patients than he?

Men have time and again tried vainly to legislate quackery out of existence—it is simply impossible. The educated physician has to compete with the ignorant and vulgar, always has had to, and probably always will, but who doubts the final issue? To argue anything but ultimate success, is to argue that the course of civilization is backward. So "Diploma's" few instances of successful ignorance may go for nothing. If I were disposed, I might cite numerous instances of honorable, intelligent, scientific men, who have attained success unaided during the time which "Diploma" has occupied in floundering, and, I fear, grumbling. In fact, "Diploma's" letter will be read by hundreds of men who will smile at what contradicts the lesson of their lives.

I believe as implicitly as "Diploma," or any one else, that the standard of medical education should be elevated, but not for his reasons I do not believe that any course of study, either at Bellevue or anywhere else, can guarantee success to any man. Medical colleges may prepare a man for his battle, and the better they prepare him the better fight he may make if he will, but his success is always his own. It may be hastened or detained by wealth, and influence; but without either, if he have the energy of a full manhood, he may attain it. I believe that the law of supply and demand alone can regulate the number of men who shall practice medicine, and that medical colleges are just as subservient to that law as individuals, and have no more control over it than individuals. Then let them do their work. Encourage them to elevate their standard for the public good, and not attempt it for private gain.

Jeremiahs will wail, but the world moves, and meritorious men will succeed in the future as they have succeeded in the past.

I do not believe the professors of our colleges to be the men whom "Diploma" alleges. That many of them are honorable, high-toned men, I know; and the imputation that they graduate men merely for the money's sake is as base as it is groundless.

To sum them up, then, "Diploma" is a disappointed man. He graduated at a first-class literary college, then at Bellevue—served eighteen months in hospital—went abroad for a year—returned—wrote articles for the papers, on which he was "complimented by many of the older members of the profession," etc., etc., made a humiliating failure of his life—tells us all about it—and falls out of our ranks blaming everybody but himself, and warning everybody to beware of his example.

I will take up his warning, and admonish every young man studying medicine, about to study it, or just commencing to practice it, to beware of his example. Beware of his failure, and make your success certain. Do not rest with the knowledge that you have an education "much more extensive than that usually enjoyed by students," and therefore think that you must succeed; but remember that with all his erudition he made a failure, while less trained men, by untiring energy and honest endeavor, have elevated themselves to places of honor and emolument.—SUCCESS. —*Medical Record.*

